# Selective Extraction of a Monoisotopic Ion While Keeping the Other Ions in Flight on a Multi-Turn Time-of-Flight Mass Spectrometer

**DOI:** 10.5702/massspectrometry.A0088

**Published:** 2020-08-20

**Authors:** Toshinobu Hondo, Hiroshi Kobayashi, Michisato Toyoda

**Affiliations:** 1 MS-Cheminformatics LLC, Toin, Inabe-gun, Mie, Japan; 2 Project Research Center for Fundamental Sciences, Graduate School of Science, Osaka University, Toyonaka, Osaka, Japan; 3 Hamamatsu Photonics K.K., Hamamatsu, Japan; 4 Department of Physics, Graduate School of Science, Osaka University, Toyonaka, Osaka, Japan

**Keywords:** time-of-flight mass spectrometer, multi-turn, ion selection, MS/MS

## Abstract

Using a multi-turn time-of-flight (TOF) mass spectrometer, we have extracted a single xenon isotope ion, ^129^Xe^+^, from its orbit at given a lap number without disturbing the rest of isotopes. After detecting the ^129^Xe^+^ at 20 laps, the rest of the xenon isotope spectrum was obtained at 30 laps, which generated a TOF spectrum where the TOF difference between ^129^Xe^+^ and ^130^Xe^+^ was 87.4 μs while ^130^Xe^+^ and ^131^Xe^+^ were 1.03 μs. The time distance between ^129^Xe^+^ and other isotopes can be set by any lap difference that is a factor of 8.7 μs, which depends on the acceleration voltage and the mass of the ion. Method accuracy was verified by comparing the isotopic abundance ratio of the xenon sample after withdrawing one of the ions from the isotope cluster to the abundance ratio obtained from the conventional method. The TOF stability was also evaluated at various lap numbers between 10 to 230.

## INTRODUCTION

The mass accuracy of the miniaturized multi-turn time-of-flight (TOF) mass spectrometer using a unique mass assignment algorithm has been reported.^1)^ The design of the multi-turn TOF mass analyzer provides an infinite flight path by keeping ions in an infinite orbit using perfect-ion focusing.^2)^ Ions can be ejected out from orbit and introduced into the detector at a given timing. Unlike reflectron TOF mass spectrometers, mass assignment for the multi-turn instrument is highly predictable due to the linear “TOF equation,”^3)^ which is advantageous for automatically ejecting a specific ion out of orbit at a desired timing by a simple analyzer control system. Using the determined “TOF equation,” the location of a given ion in the analyzer at a known time can be calculated quickly and precisely. Therefore, it is possible to eject and detect an ion at a given lap number and then introduce the remaining ions into the detector some laps later. Such a control protocol can generate a series of TOF difference between given ions, which is a powerful tool to investigate the microchannel plate gain drop issue after intense ion flux detection^4)^ by using ion. Furthermore, we can hold an ion cluster keep flying and extract one by one at a given timing that may be a good tool for the first stage analyzer on the tandem TOF mass spectrometry (TOF/TOF).

Xenon is a good sample for a spectral profile testing since it has a unique isotope distribution. As shown in Table 1, xenon has several abundant isotopes, such as ^129^Xe, ^131^Xe, and ^132^Xe. The abundance ratios of terrestrial air xenon are 0.983 and 0.787 for ^129^Xe/^132^Xe and ^131^Xe/^132^Xe, respectively, so mass spectral peak intensities for ^129^Xe^+^ and ^132^Xe^+^ are nearly equal as of only 1.7% difference. Therefore, the accuracy of monoisotopic peak selection without disturbing other ions can be evaluated by monitoring the above isotope ratios and their respective TOF values.

**Table 1:** Table 1. Xenon isotopes and their relative abundances.^11)^

Isotope	Mass	Isotopic abundance
^124^Xe	123.9058930	0.0009
^126^Xe	125.904274	0.0009
^128^Xe	127.9035313	0.0192
^129^Xe	128.9047794	0.2644
^130^Xe	129.9035080	0.0408
^131^Xe	130.9050824	0.2118
^132^Xe	131.9041535	0.2689
^134^Xe	133.9053945	0.1044
^136^Xe	135.907219	0.0887

## EXPERIMENTAL

### Instrumentation

The miniaturized multi-turn time-of-flight (TOF) mass spectrometer^5)^ infiTOF-UHV (MSI.TOKYO, Inc., Tokyo, Japan) was used with in-house modifications reported previously.^1,6)^ Ions were detected by MIGHTION (Hamamatsu Photonics K.K., Hamamatsu, Japan),^7)^ which is a micro-channel plate (MCP) combined with an avalanche diode. The detector signal was passed through a model C11184 preamplifier (Hamamatsu Photonics K.K., Hamamatsu, Japan), followed by waveform acquisition using an Acqiris U5303A 1 GS *s*^−1^ high-speed digitizer (Acqiris, Geneva, Switzerland).

The detector operation conditions are 350 V for avalanche diode voltage, −4.96 kV for MCP-In potential, and 560 V for between MCP-In and MCP-Out. Xenon gas (Takachiho Chemical Industrial Co., Ltd.) was introduced into the electron ionization (EI) chamber using a 1 m length of 0.1 mm inner diameter PEEK (Polyether Ether Ketone) capillary tubing. The vacuum condition in the ionization chamber during sample measurement was 3.4×10^−3^ Pa (The pressure when the sample introduction valve was closed was 2.2×10^−4^ Pa). Ionization energy was set to 30 eV and filament current to 3200 mA for xenon profile spectrum monitoring, or 2880 mA for xenon ion counting experiments.

### Analyzer timings

**Figure 1:**
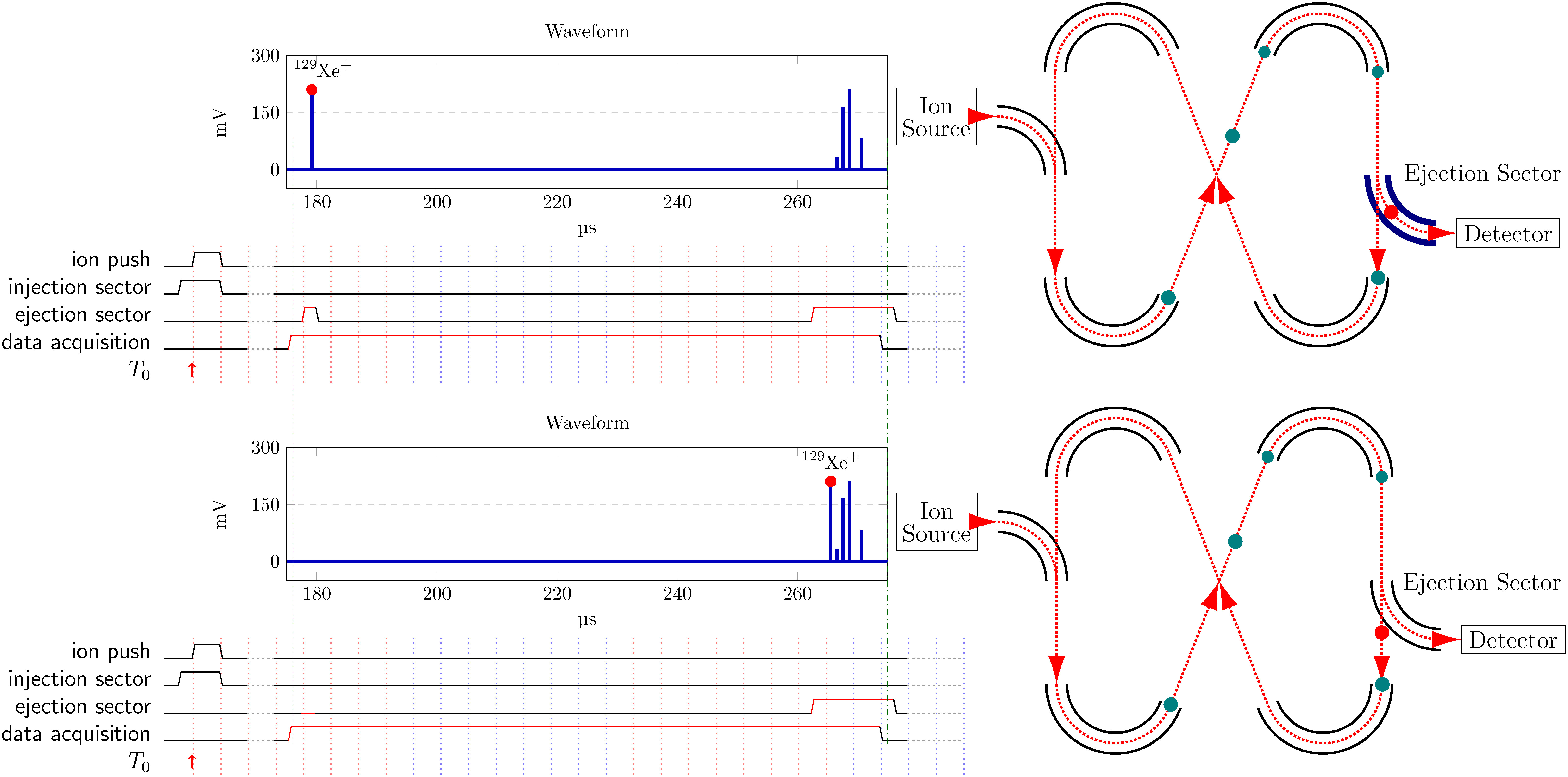
Fig. 1. Sector control timing schematics.

Figure 1 illustrates the relationship between analyzer timings and the detector signal. The conventional timing for acquiring a xenon spectrum is illustrated in the bottom of Fig. 1, where the infiTOF is operating in multi-turn mode, and the ejection sector at the halfway point of the orbital path is closed for the sample orbital period until the xenon ion cluster arrives after 30 laps.

One additional functionality was added to the control system, which allows additional, precise ejection sector timing. Figure 1 top illustrates selecting and ejecting ^129^Xe^+^ at 20 laps, while the rest of the control timings remain the same. In this case, we have a xenon TOF spectrum at 30 laps with the absence of ^129^Xe^+^.

The ejection sector has consisted of the high-voltage MOSFET push–pull mode switch,^8)^ which both rise and fall time is about 100 ns.

### Procedure

**Figure 2:**
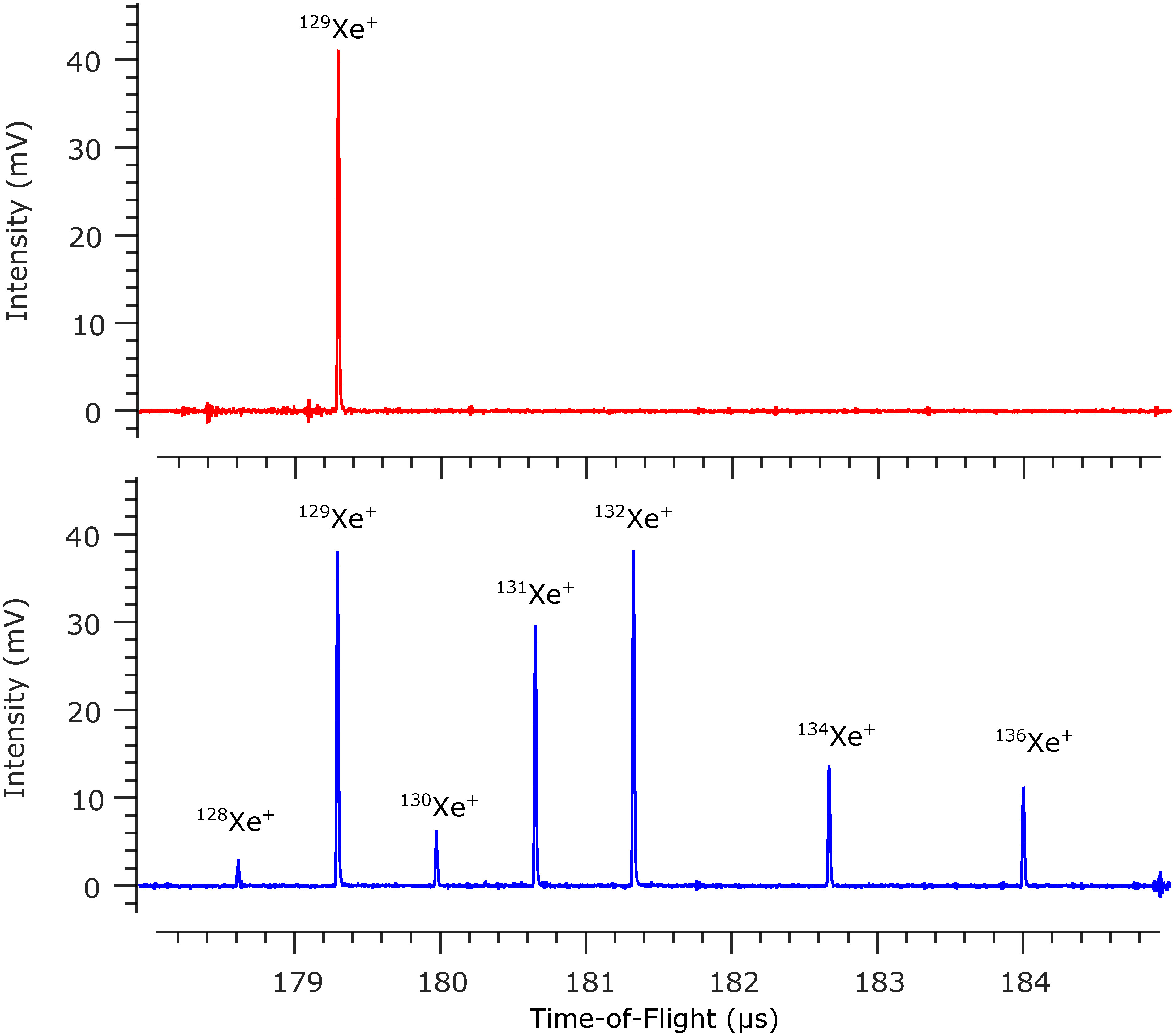
Fig. 2. Xenon isotope spectrum (bottom), and ^129^Xe^+^ at 20 laps after narrowing down ejection timing (top).

After setting analyzer conditions and experimental system equilibrations were set, mass assignment needs to be verified using data from 20 and 30 laps of ^132^Xe^+^.^1)^ The data acquisition software determines the timings for the ejection sector for a given chemical formula and lap number by using the “TOF equation” determined from the mass assignment process. To obtain the TOF spectrum of xenon at 20 laps, two parameters were set in the software: the formula for ^131^Xe^+^ and the number of laps to 20, which produced the spectrum shown in Fig. 2 bottom. At this point, the software adjusts the timing for ^131^Xe^+^ to be the center of the region of interest for the xenon isotope cluster. After this spectrum is visible on the real-time monitor, the duration parameter can be narrowed down for the ejection sector timing until single ^129^Xe^+^ peak is obtained without any other ions visible on the spectral monitor screen as shown in Fig. 2 top. The timing for the ejection sector was determined to be open at 176.03 μs for 0.7 μs, which means that ^129^Xe^+^ is always passing through the ejection sector during this time frame as long as “TOF equation” does not change. A second ejection sector timing was added to the software, which can either be enabled or disabled during each acquisition protocol. Using this modification, ^129^Xe^+^ at 20 laps can be monitored while acquiring the remaining xenon spectrum at a minimum of 21 laps or higher by setting the above timing values into the second ejection sector parameter and enabled.

## RESULTS AND DISCUSSION

### Effect of ejection-sector switch to the ion abundance ratio

**Figure 3:**
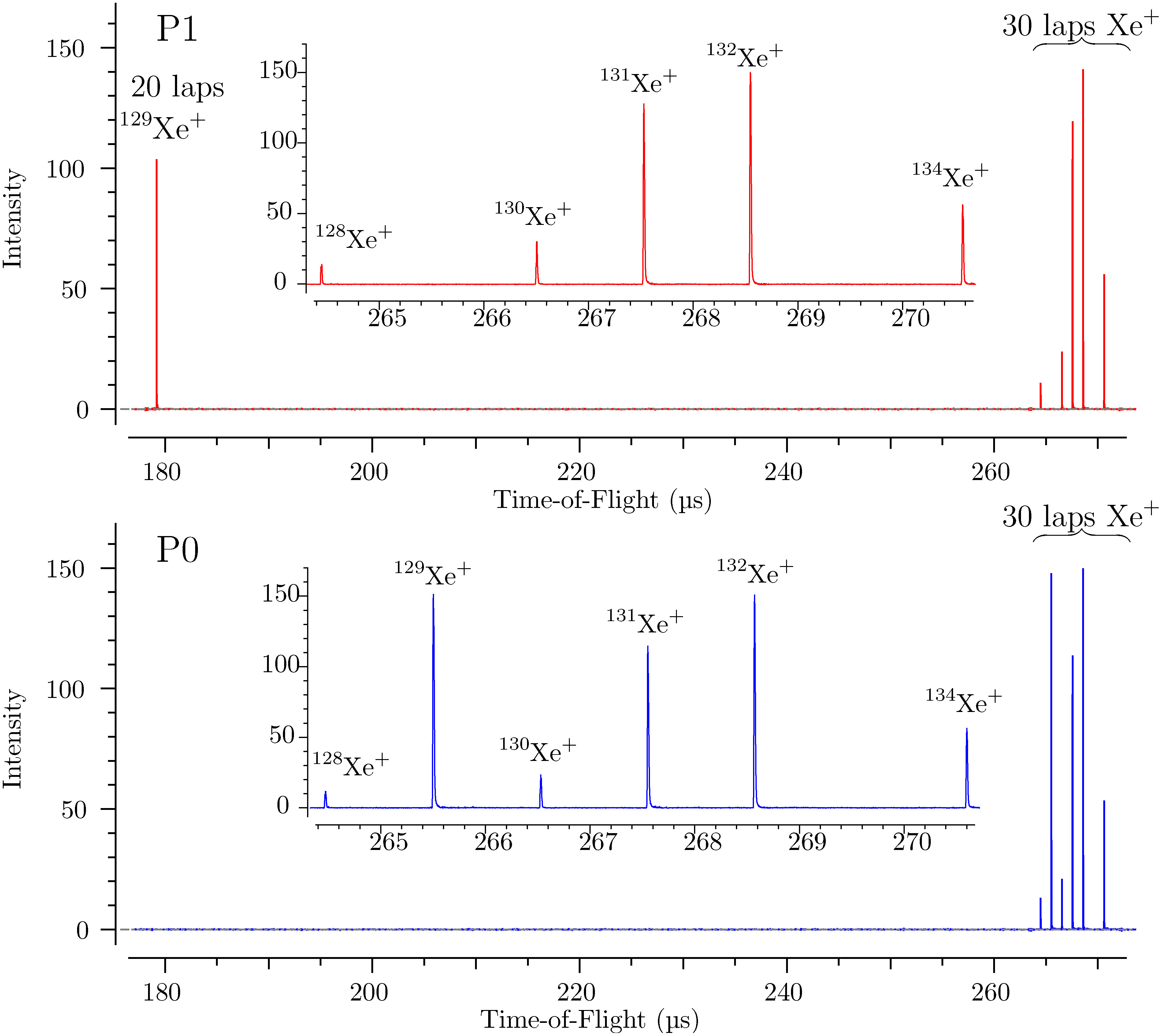
Fig. 3. The P0 spectrum represents protocol 0, where no ^129^Xe^+^ ions were ejected early (conventional multi-turn TOF spectrum of Xe isotopes). The P1 spectrum represents protocol 1, where ^129^Xe^+^ was withdrawn at 20 laps and monitored by the detector.

Table 2 shows the abundance ratio of xenon isotopes acquired from 30 laps of xenon. The count rate for ^132^Xe^+^ was adjusted to 36.3%, where 36.3 ^132^Xe^+^ ions were counted of 100 ion push triggers. “Protocol 0” (P0) is acquiring a xenon spectrum at 30 laps with our normal monitoring setup; “protocol 1” (P1) is also acquiring a xenon spectrum at 30 laps, but ^129^Xe^+^ was ejected and monitored at 20 laps. These two protocols were alternated for every ion push trigger event. As shown in the Table 2, the isotope abundance ratio for ^130^Xe^+^ and ^131^Xe^+^ against ^132^Xe^+^ showed excellent agreement between P0 and P1. The obtained counts for 20 laps of ^129^Xe^+^ is lower than 30 laps on Table 2, which is instrument tuning dependent, however, the obtained count ratios within the same lap numbers were stable. We can conclude that the ^129^Xe^+^ was successfully ejected from a cluster of xenon isotopes without affecting the abundance ratio, *i.e.*, no ion loss was observed by removing it from a sample cluster. Figure 3 shows the corresponding spectra for P0 and P1. The spectra presented in this paper uses ejection-sector open duration time of 700 ns, however, spectral profile change did not observe until it narrows down to 640 ns (data were not shown). Therefore, any ion peak, where the TOF away from at least 640 ns from adjacent ion peaks, can be withdrawing without disturbing other ions by using this method.

**Table 2:** Table 2. Comparison of xenon isotope abundance ratio by using ion counting.

Protocol 0 (30 laps xenon)						
Formula	*m*/*z*	Counts	[Xe]/[^132^Xe]	Time-of-flight(μs)		
^129^Xe^+^	128.9048	21038	0.996	265.5132		
^130^Xe^+^	129.9035	3390	0.161	266.5388		
^131^Xe^+^	130.9051	16569	0.784	267.5635		
^132^Xe^+^	131.9041	21122	1.000	268.5817		
^134^Xe^+^	133.9054	8310	0.393	270.6103		
Protocol 1 (30 laps xenon, withdraw ^129^Xe^+^ at 20 laps.)						
Formula	*m*/*z*	Counts	[Xe]/[^132^Xe]	%error	Time-of-flight(μs)	δ*t*
^129^Xe^+^	(128.8996)	(11627)	n/a	n/a	179.1874	n/a
^130^Xe^+^	129.8987	3558	0.158	1.55%	266.5391	0.31
^131^Xe^+^	130.8986	17756	0.789	0.54%	267.5642	0.68
^132^Xe^+^	131.8956	22512	1.000	n/a	268.5824	0.68
^134^Xe^+^	133.8929	8908	0.396	0.59%	270.6107	0.33

Protocol 0: count ratio for 30 laps of xenon; Protocol 1: count ratio for 30 laps xenon, but withdraw ^129^Xe^+^ at 20 laps. Total 60055 ion push triggers were acquired.

### The *m*/*z* accuracy at various numbers of laps

**Figure 4:**
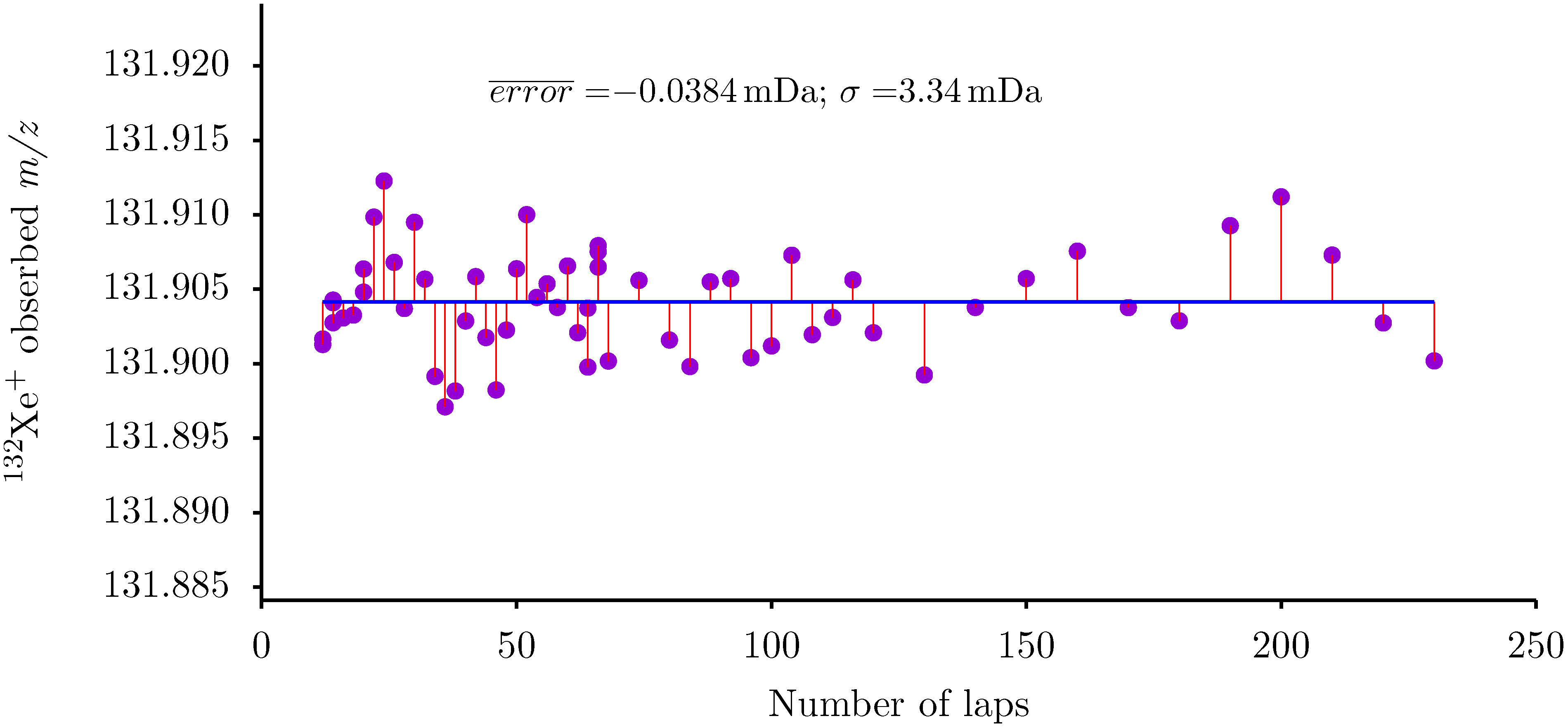
Fig. 4. Deviations of TOF and *m*/*z* from the TOF equation calculated values (horizontal line) at a various number of laps.

Figure 4 shows the mass errors for ^132^Xe^+^ at various numbers of laps. The standard deviation of mass error was 3.32 mDa, and the mass resolving power at 20 and 200 laps were 8,700 and 40,700, respectively. The standard deviation of time errors compared to the TOF computed using the “TOF equation” for ^132^Xe^+^ in the range of 94.0 to 2016.7 μs was approximately 8.4 ns, which is two orders of magnitude lower than the duration of the ejection sector was open. Both *m*/*z* and TOF for ^132^Xe^+^ stayed the same between protocols, whether the ^129^Xe^+^ was withdrawn at 20 laps. This demonstrates how closely the ion flight path follows the “TOF equation,” and that ion flight is not disturbed by the withdrawal of a monoisotopic ion during TOF separation in the analyzer.

### Capability of the method for the TOF/TOF application

The results shown here also suggests that this analyzer is an excellent candidate as a first mass analyzer in a TOF/TOF instrument^9)^ with the capability of selecting multiple precursor ions from the single ion push cycle. In the case of ultra high-performance liquid chromatography (UHPLC) analysis of complex sample matrices, such as protein and drug metabolites identification, many single chromatographic peaks consist of multiple compounds. The peak width on a UHPLC chromatogram, in general, is approximately one second or less, and about 10 sample points per peak are required to determine chromatographic peak area effectively. So it is challenging to apply data-dependent TOF/TOF for multiple components in a single peak, which has led to investigation of several non-tandem TOF approaches^10)^. However, if the analytes are more than one dalton *m*/*z* difference in an ion trigger cycle, it is possible to select one precursor ion to introduce to the second mass analyzer (MS2) and keep the other ions in the multi-turn analyzer (MS1). The orbital period is mass-dependent, corresponding to the TOF for the length of the figure-eight orbit (0.662 13 m), *e.g.*, 13.23 μs and 13.45 μs for *m*/*z* 300 and 310 ions, respectively. Assuming ions of *m*/*z* 300 and 310 co-exist in a spectrum, *m*/*z* 300 could be ejected into MS2 at an early lap number, and then wait for an additional 134.5 μs (10 more laps) to introduce *m*/*z* 310 into MS2.

## CONCLUSION

New, unique ejection sector control capabilities were added to the multi-turn TOF mass spectrometer to extract ^129^Xe^+^ from a xenon isotope cluster. Setting a 700 ns duration for the ejection-sector shows excellent results for monitoring the ^129^Xe^+^ peak at 20 laps while the rest of the isotopes remain in orbit for subsequent measurement at 30 laps. The peak abundance ratio obtained from xenon isotopes at 30 laps, both with and without ^129^Xe^+^ shows less than 5% error on the profile spectrum under rich ion flux conditions. The accuracy of the abundance ratio determined by ion counting shows less than 1.55% error for ^130^Xe^+^ and less than 0.59% error for 131 and 132 xenon.

We also investigated the *m*/*z* errors at various laps from 10 to 230. Since the mass assignment method is a linear equation, mass error is reflecting the TOF fluctuations directly. The standard deviation of *m*/*z* error for 10 to 230 laps was 3.32 mDa, which corresponds to 8.4 ns on average, which is accurate enough to select an ion by controlling the ejection sector. The *m*/*z* accuracy was not affected by the use of the ejection sector for ion selection. Use of the ejection sector did not affect either peak intensity or *m*/*z* errors, where the *m*/*z* error is the TOF conformity to the “TOF equation” on this instrument.

## Notes

Mass Spectrom (Tokyo) 2020; 9(1): A0088
